# A Rare Case of Transient Acantholytic Dermatosis (AKA. Grover’s Disease) with Concomitant Pediculosis Pubis: An Atypical Presentation and First Documented Case Report

**DOI:** 10.3390/dermatopathology8040052

**Published:** 2021-10-22

**Authors:** Gehan A. Pendlebury, Peter Oro, Drew Merideth, Eric Rudnick

**Affiliations:** 1 College of Osteopathic Medicine, Nova Southeastern University, Fort Lauderdale, FL 33314, USA; 2 School of Osteopathic Medicine in Arizona, A.T. Still University, Mesa, AZ 85206, USA; peter.oro94@gmail.com (P.O.); dmerideth3@icloud.com (D.M.); 3 Department of Dermatology, College of Medicine, University of Florida, Gainesville, FL 32610, USA; info@dazzlingderm.com

**Keywords:** Grover’s disease, acantholytic dermatosis, pediculus pubis, dermatopathology

## Abstract

A 66-year-old male presented with a one-month history of persistent pruritic eruptions distributed mainly on the trunk. A punch biopsy from the left upper abdomen revealed focal acantholytic dyskeratosis with mixed inflammatory infiltrate in the dermis composed of numerous eosinophils. Grover’s disease was diagnosed based on the clinical and histopathological findings. Appropriate treatment was initiated but failed to relieve symptoms of itchiness. A further investigation of the hair follicles under mineral oil preparation revealed an infestation of pediculosis pubis. Subsequent treatment with Ivermectin and permethrin cream led to the complete resolution of his symptoms. This case report highlights an unusual and first documented case of Grover’s disease with a concomitant infestation of pediculosis pubis. To date, no reported cases in the literature have associated Grover’s disease with pubic lice infestation. However, there are three reported cases of concurrent scabies and Grover’s disease in the literature. This rare case underscores the clinical value in further investigating treatable underlying conditions in patients with suspected transient acantholytic dermatosis.

## 1. Introduction

Originally described by Ralph Grover in 1970, transient acantholytic dermatosis, also known as Grover’s Disease, is a rare dermatologic condition of unknown etiology characterized by an eruption of 2–5 mm erythematous, pruritic papules typically found on the trunk and proximal extremities [1]. Grover’s Disease commonly presents in middle- to upper-aged white males and resolves in weeks, although many times cases are chronic, recurring, and treatment-resistant. Four subtypes of acantholytic dermatosis have been described with varying patterns of acantholysis. The histology demonstrates areas of acantholysis with dyskeratosis, diagnostic features of the disease. [2]. While it should always be considered a differential diagnosis by clinicians, this condition has been shown to be quite rare. A study done by Sterit et al. in Berne in 1997 and 1998 demonstrated that 21 cases of Grover’s disease were histologically diagnosed from over 300,000 skin biopsies [3].

A much more common dermatologic condition observed worldwide and costing Americans roughly $350 million in treatment per year is louse infestation. There are three species of lice parasitic to humans—pediculus humanus (body lice), pediculus humanus capitis (head lice), and pediculosis pubis (pubic lice)—with head lice occurring most commonly [4]. Worldwide, hundreds of millions of individuals across all socioeconomic statuses are infected each year [4]. Pubic lice are typically spread through sexual intercourse, infecting the pubic, groin, and perianal regions. However, pediculosis pubis has been known to infect other areas such as the chest, abdomen, beard, or axilla. We herein present the first documented case of transient acantholytic dermatosis with concomitant pediculosis pubis.

## 2. Case Report

The patient is a 66 year old male with a past medical history of hypertension who presented to the clinic for the evaluation and management of a persistent pruritic eruption for one month, distributed on the trunk. The patient denied any contacts with similar itching. Many over-the-counter anti-pruritic products were ineffective.

**Figure 1:**
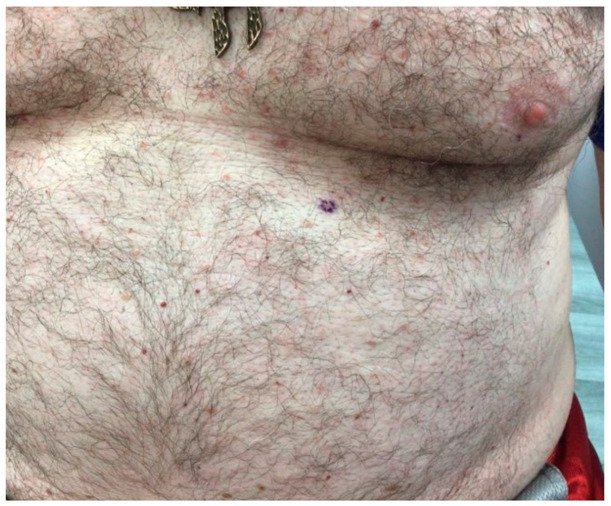
On a background of normal skin there are scattered small pink-to-erythematous papules on the chest and upper abdomen.

Clinical examination revealed multiple erythematous 2–4-mm pruritic papules mainly on the trunk (Figure 1). The patient was clinically diagnosed with transient acantholytic dermatosis, and clobetasol propionate 0.05% ointment was initiated. 

**Figure 2:**
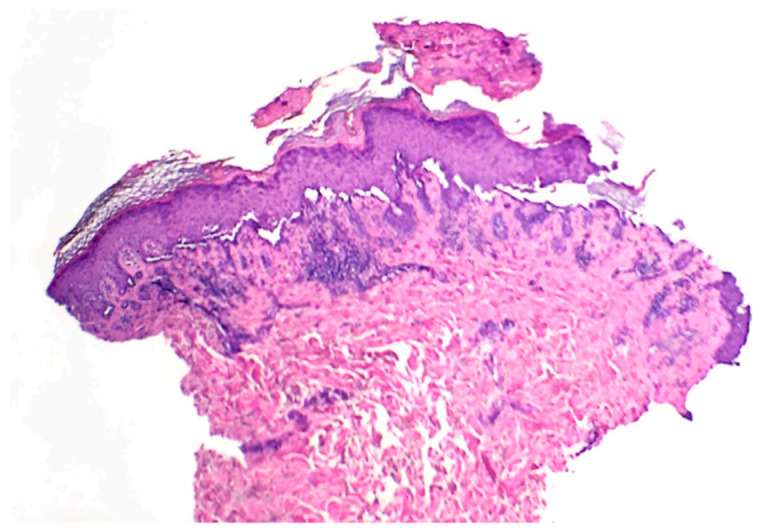
Skin punch biopsy specimen displaying superficial crusting with basilar acantholysis (40×).

**Figure 3:**
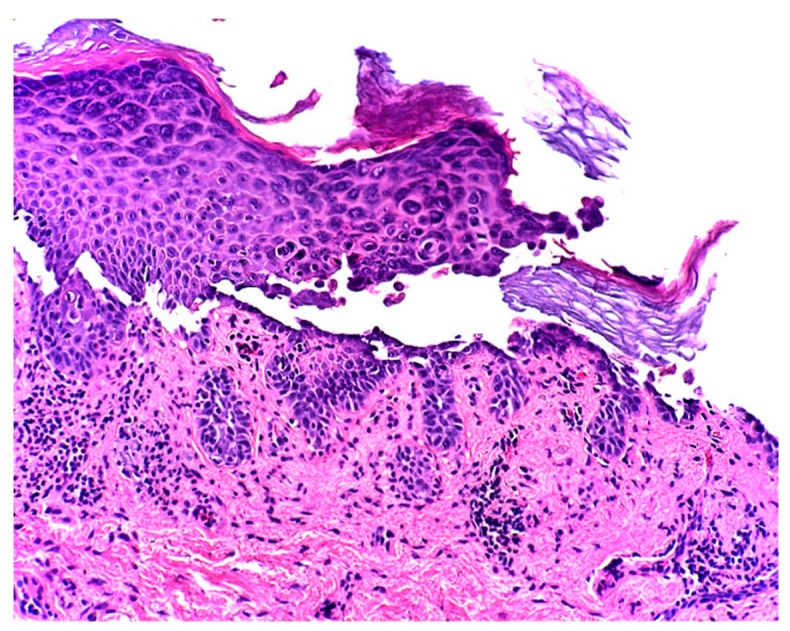
The epidermis showed suprabasilar acantholysis with dyskeratosis. Within the superficial dermis there is a lymphohistiocytic infiltrate with few eosinophils (200×).

At the two-week follow-up evaluation, a 4-mm punch biopsy was performed on the left upper abdomen due to persistent pruritus despite the clobetasol propionate treatment. The biopsy revealed focal acantholytic dyskeratosis with a mixed inflammatory infiltrate in the dermis beneath with numerous eosinophils, consistent with the diagnosis of Grover’s disease (Figure 2 and Figure 3). 

**Figure 4:**
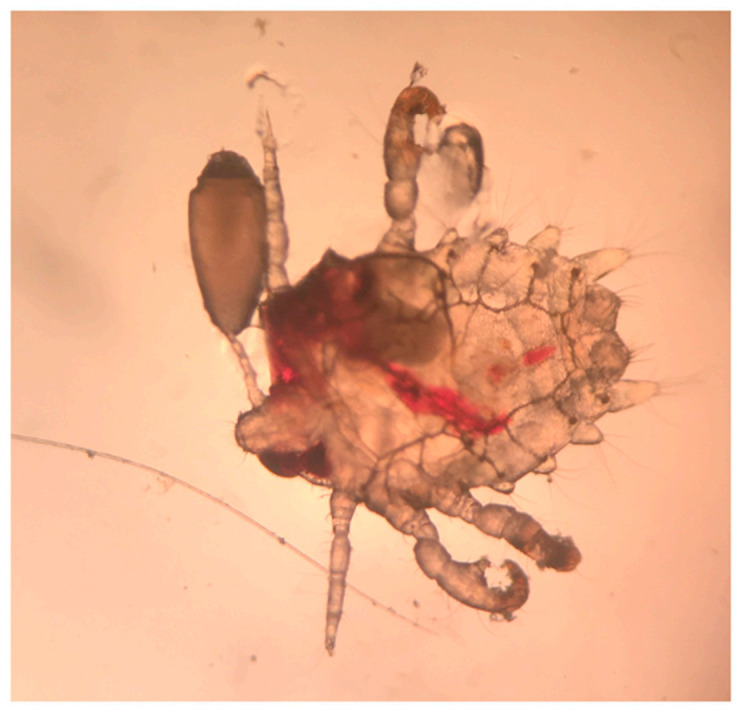
Pediculosis pubis (also known as “crabs” and “pubic lice”) with recent blood meal and adjacent egg (100×).

Two weeks after the punch biopsy, the patient returned for suture removal and due to persistent itching still without any relief. At this visit, a closer examination revealed concretions at the base of the truncal hair follicles. A sample of these clinical concretions under a mineral oil preparation revealed pediculosis pubis (Figure 4). The patient was treated with Ivermectin (200 mcg/kg) and Permethrin cream once with repeat therapy after seven days. His itching resolved after the successful treatment of crab lice. 

## 3. Discussion

The patient’s clinical presentation and the histopathological findings of the skin were consistent with transient acantholytic dermatosis. However, a two-week treatment with a clobetasol propionate 0.05% ointment did not resolve the symptoms. Skin scrapings at a later visit demonstrated minute concretions which under a mineral oil preparation revealed pediculosis pubis.

In the initial visit, a dermatoscope could have been used to identify and select appropriate lesions for biopsy in this patient. However, this was decided to be clinically unnecessary since lesions on the patient’s trunk were conspicuous. Nevertheless, a dermatoscope has a significant clinical utility in improving the recognition of lesions associated with Grover’s disease. Such lesions are sometimes difficult to visualize with the naked eye. A dermatoscopic central star-like pattern of brown scales has been shown to be helpful in the diagnosis of this condition [5,6].

At the time of this writing, this is the first reported case of a concomitant skin infection involving acantholytic dermatosis and pediculosis pubis. Three cases in the literature describe an association between Grover’s disease and scabies caused by S. scabiei [7,8,9]. However, there are no reported cases that show a concomitant skin infection with pediculosis pubis and Grover’s disease. 

The cases that have been reported had a very similar presentation of a pruritic skin eruption associated with histopathological findings consistent with Grover’s disease and scabies. Treatment for scabies for the three patients led to a complete resolution of the pruritus, which further elucidates a possible role of S. scabiei in the pathogenesis of some cases of Grover’s disease. 

The histopathology of our patient showed that the epidermis contained foci of acantholysis and dyskeratosis with mixed inflammatory infiltrates in the dermis beneath with numerous eosinophils. These findings are consistent with Grover’s disease [10]. A study published in 1999 by Davis et al. demonstrated that 22% of Grover’s disease cases presented with an eosinophilic infiltrate in their skin biopsies [10]. In contrast, the majority of cases of lice infections present with eosinophilia [11]. Furthermore, it remains unclear whether the eosinophilic infiltrate in the skin biopsy of our patient was due solely to Grover’s disease, to the lice infestation, or to both pathological mechanisms. Treatment with Ivermectin and permethrin cream resolved his itching, indicating that pediculosis pubis may have played a role in the pathogenesis of Grover’s disease in this particular case.

The oral and topical formulations of Ivermectin have been shown to be effective in killing head lice [12]. Ivermectin has multiple mechanisms of action that make it very effective as an antiparasitic and as an anti-inflammatory agent. Ivermectin selectively binds and opens Glutamate-gated chloride channels (GluCls) found in the muscle nerves of helminths and parasites, which leads to the hyperpolarization and paralysis of the lice [13]. Additionally, the anti-inflammatory mechanism of Ivermectin works by inhibiting the production of lipopolysaccharide (LPS)-induced inflammatory cytokines such as tumor necrosis factor-a (TNF-a), interleukin-1ß (IL-1ß) and interleukin-6 (IL-6). Ivermectin also stimulates the production of the anti-inflammatory cytokine interleukin-10 (IL-10) [14].

The etiology for Grover’s disease remains unknown. However, there are postulated mechanisms for the pathogenesis of the disease. One proposed mechanism is related to sweating, whereby there is a blockage of the eccrine sweat glands, which leads to the leakage of proteolytic enzymes that are contained within the obstructed gland. These enzymes can potentially cause inflammatory infiltrate and acrosyngeal acantholysis [15].

On the contrary, a study done by Scheinfeld et al. revealed that more cases of Grover disease were reported in the winter season when compared to the summer season, specifically in elderly men with xerosis cutis [16]. This study concluded that the cause of Grover’s disease was unlikely to be due to sweating and was more likely correlated to impaired epidermal integrity [16]. More research is needed in this area for a deeper understanding of the underlying etiology of acantholytic dermatosis.

Despite the uncertainty of the etiology, multiple risk factors and triggers which have been associated with acantholytic dermatosis have been reported in the literature. Such triggers include:

- Ionizing radiation [17]
- Mechanical irritation [18]
- Solid organ transplant [19,20]
- Scabies [7,8,9]
- Atopic dermatitis, contact dermatitis, and xerosis cutis [21]
- Medications such as:
  ○ Ribavirin [22]
  ○ Anastrozole [23]
  ○ Interleukin [4,24]
  ○ Cetuximab [25]
  ○ BRAF inhibitors (e.g., vemurafenib, dabrafenib) [26]
  ○ Immune checkpoint inhibitors [27]

Many of the listed conditions are challenging to detect, identify, and treat when compared to this particular case of pediculosis pubis. It remains unclear whether in our case Grover’s disease developed prior to or after the pediculosis pubis infestation. However, considering the resolution of symptoms following the targeted treatment of the lice, it remains to be seen whether the lice could have played a role in provoking the disease process. Although pediculosis pubis has not been associated as a trigger with Grover’s disease, it warrants further research investigation.

## 4. Conclusions

In conclusion, we present a rare case of a concurrent infestation of pediculosis pubis with acantholytic dermatosis. Such a case has never been reported in the literature. Likewise, lice are typically not associated with Grover’s disease and are therefore not considered to be a potential trigger. However, it is not unusual for Grover’s disease to present with another easily treatable underlying disorder such as the one in this case. As such, a thorough investigation of an underlying trigger in cases of Grover’s disease is warranted by physicians. If a trigger is discovered, an appropriate prompt treatment may provide rapid relief for the patient and yield successful clinical outcomes.
